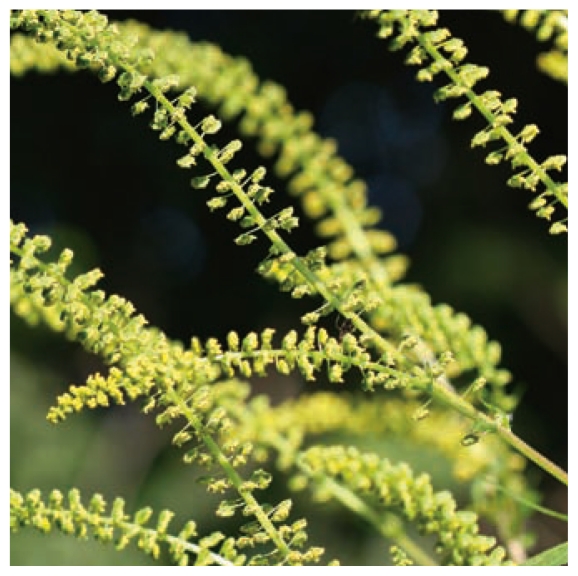# The Beat

**Published:** 2011-04

**Authors:** Erin E. Dooley

## DOI to Study Chukchi Oil Spill Impact

As part of a court-ordered supplemental review of oil and gas leasing off Alaska’s northwest coast, the Department of the Interior will assess potential environmental impacts of a major oil spill in the Chukchi Sea.^1^ The court order resulted from a summer 2010 ruling that found the department had not properly analyzed the environmental impacts of natural gas development related to a 2008 lease sale of 2.8 million acres in the area. A revised draft of the environmental impact statement should be ready by summer 2011 and will be open for 45 days for public comment, with the final review expected by October.

**Figure:**
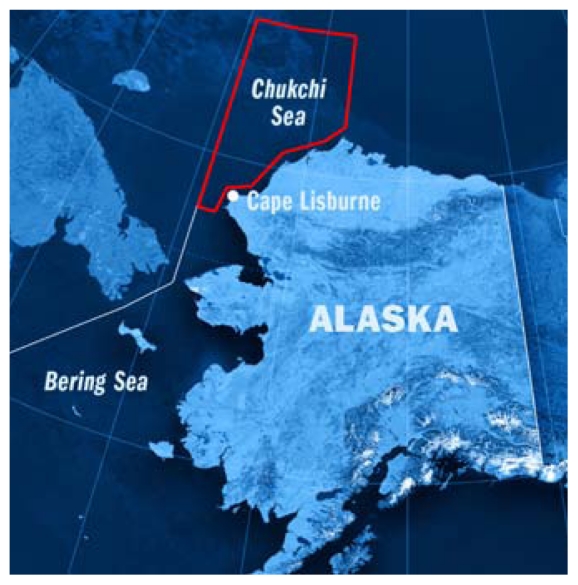


## PBDEs Off Wal-Mart Shelves

Beginning in June 2011 Wal-Mart will implement testing measures to verify compliance with its ban on polybrominated diphenyl ether (PBDE) flame retardants in certain products on its shelves.^2^ Wal-Mart’s efforts come without any federal regulation on the compounds, which only recently have begun to be regulated at the state level. Sampling during the National Health and Nutrition Examination Survey 2003–2004 revealed PBDEs in the bodies of nearly every participant tested.^3^ Although health effects data are still limited for these compounds, their persistence and ubiquity have raised substantial scientific concern.

**Figure:**
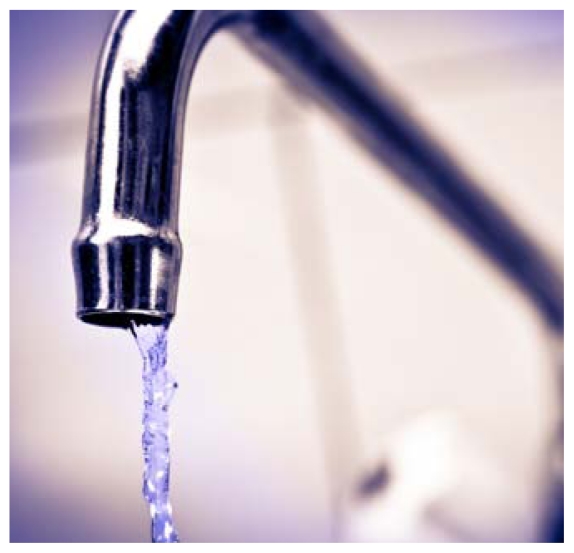


## EPA Proposes Third Unregulated Contaminant Monitoring Reg

The U.S. EPA recently proposed monitoring for 30 currently unregulated drinking water contaminants, including 28 chemicals and 2 viruses.^4^ The monitoring data would provide information about the prevalence of contaminants on the list to support future EPA decision making. Among the chemical contaminants are several perfluorinated compounds, endogenous and exogenous hormones, and metals; 1,3-butadiene, a human carcinogen; and 1,2,3-trichloropropane, an animal carcinogen. The public may comment on the proposed list through 2 May 2011.

## Higher Latitudes See Longer Ragweed Season

One significant cause of seasonal allergies is plants from the genus *Ambrosia*, which includes several types of ragweed. A study of ragweed pollen data from 10 U.S. and Canadian sites shows the duration of the pollen season increased by up to 27 days since 1995 at latitudes above about 41°N.^5^ Papillion, Nebraska, at 41.15°N, has a season 11 days longer than in 1995, Minneapolis, Minnesota (45.00°N), has a season 16 days longer, and Saskatoon, Saskatchewan (52.07°N), has a season 27 days longer. An estimated 10% or more of the U.S. population is sensitive to ragweed pollen, and by one estimate allergies cost the United States approximately $21 billion per year.^5^

## Protests against New Asbestos Plant in India

Construction on an asbestos manufacturing plant in the Indian state of Bihar has come to a halt after six months of student-led protests, according to news reports from the subcontinent.^6^ Several dozen countries now ban most or all forms of asbestos,^7^ and earlier this year, the Collegium Ramazzini reintroduced its call for a global ban on asbestos.^8^ An estimated 125 million people are exposed to asbestos in the workplace, and thousands of deaths and new diagnoses of asbestos-related disease are reported each year.^8^

**Figure:**